# The genetic basis for adult-onset idiopathic dilated cardiomyopathy in people of African descent

**DOI:** 10.1007/s10741-023-10302-9

**Published:** 2023-03-14

**Authors:** Nqoba Tsabedze, Michele Ramsay, Amanda Krause, Quinn Wells, Dineo Mpanya, Pravin Manga

**Affiliations:** 1 Division of Cardiology, Department of Internal Medicine, School of Clinical Medicine, Faculty of Health Sciences, University of the Witwatersrand, Charlotte Maxeke Johannesburg Academic Hospital, 17 Jubilee Road, Parktown, Johannesburg, Gauteng 2193 South Africa; 2 Sydney Brenner Institute for Molecular Bioscience, Faculty of Health Sciences, University of the Witwatersrand, Johannesburg, South Africa; 3 Division of Human Genetics, National Health Laboratory Services and School of Pathology, Faculty of Health Sciences, University of the Witwatersrand, Johannesburg, 2001 South Africa; 4 Division of Cardiovascular Medicine, Department of Medicine, Vanderbilt University Medical Center, Nashville, 37232 TN USA

**Keywords:** Heart failure, Genetics, Idiopathic dilated cardiomyopathy, Familial dilated cardiomyopathy, Sub-Saharan Africa, Monogenic

## Abstract

Cardiomyopathies are a heterogeneous group of cardiac muscle disorders that result in dilated, hypertrophic, or restrictive pathophysiological entities. Dilated cardiomyopathy (DCM) is the most common form in sub-Saharan Africa (SSA). However, population-specific research studies reporting the actual burden of DCM in this region are still lacking. Also, little is known about the genetic basis of DCM in this population, and genetic testing is still not readily accessible. This review describes the common pathogenic genes implicated in DCM globally and discusses the evidence-based management of patients with DCM. We also present a summary of studies describing genes implicated or associated with DCM in patients residing in SSA.

**Supplementary Information:**

The online version contains supplementary material available at 10.1007/s10741-023-10302-9.

## Introduction

Dilated cardiomyopathy (DCM) is Africa’s second most common cause of heart failure, preceded only by hypertensive heart diseases [1]. Although DCM is prevalent in Africa, little is known about the genetic aetiology or mutations responsible for monogenic forms of this type of cardiomyopathy. In clinical practice, most patients are given a working diagnosis of idiopathic dilated cardiomyopathy (IDCM) without any formal genetic testing. This is partly because of limited access to genetic testing and the lack of cardiomyopathy gene panel tests based on data on genetic mutations found in African patients with DCM.

Population-based data on the burden of IDCM in Africa are lacking. In a post-mortem analysis of 90 subjects with underlying cardiovascular disease in South Africa, 17% had idiopathic DCM [2]. In a prospective registry of heart failure cases in a tertiary-level hospital in Johannesburg involving 5328 patients, 9.4% had a primary diagnosis of IDCM [3]. Although DCM is common, patients in low- and middle-income countries still present with advanced heart failure symptoms at a younger age than those in high-income countries [4, 5]. In this review, we discuss the mutational profile of DCM genetics and provide clinical guidance to clinicians managing DCM patients.

### Definitions

Cardiomyopathies are a heterogeneous group of cardiac muscle disorders associated with cardiac dysfunction. Therefore, the terminology used to define dilated cardiomyopathies varies and may overlap (Table 1).

**Table 1:** Definitions for dilated cardiomyopathies

	Definition
Dilated cardiomyopathy	Left ventricular or biventricular dilatation and systolic dysfunction not explained by abnormal loading conditions or coronary artery disease sufficient to cause global systolic impairment [6]
Genetic dilated cardiomyopathy	One or more defined genetic mutations causing dilated cardiomyopathy
Familial dilated cardiomyopathy	An individual diagnosed with idiopathic dilated cardiomyopathy (IDCM) in a family with at least: (i) two or more first-degree relatives with IDCM *or* (ii) unexplained sudden cardiac death before the age of 35 years [7]
Sporadic dilated cardiomyopathy	Idiopathic dilated cardiomyopathy in individuals *without* family members who meet the familial dilated cardiomyopathy criteria [8]
Idiopathic dilated cardiomyopathy	Dilated cardiomyopathy without any identifiable cause after excluding all potential causes, *including* genetic mutations

### Clinical manifestation

The onset of heart failure in sub-Saharan African patients generally occurs at a younger age, thus affecting the economically active group [4]. In the early stages, some patients may remain asymptomatic, whilst in others, the nonspecific symptoms of heart failure may easily be misinterpreted as related to exhaustion or pulmonary diseases. Individuals with DCM may present with acute or gradual onset of heart failure symptoms. In some instances, individuals with DCM may also present with conduction abnormalities and sudden cardiac death.

### Genetic determinants and mechanisms of genetic dilated cardiomyopathy

Globally, more than 50 genes that play a role in DCM have been identified, with some included in commercially available gene testing panels [9]. Of these genes, twelve have a definitive or strong relationship with DCM, seven had moderate evidence, and 25 had a limited role in DCM. In addition, seven have been disputed or assigned as having no known disease relationship due to the lack of evidence in human studies [9].

**Fig. 1:**
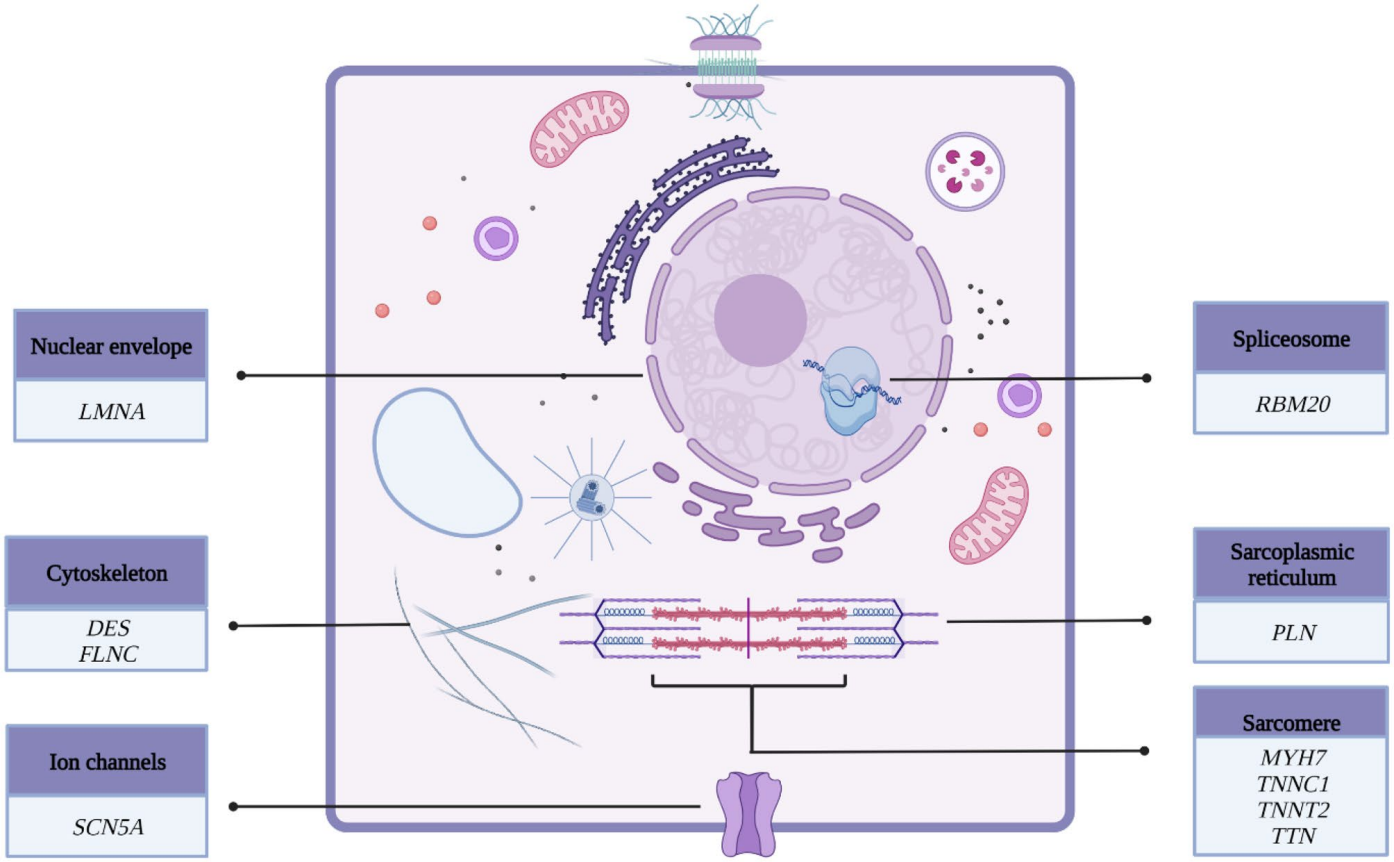
Genes with a definitive or strong relationship with dilated cardiomyopathy grouped according to their location in cardiac myocytes. *DES* desmin, *FLNC* filamin C, *LMNA* lamin A/C, *MYH7* myosin heavy chain 7, *PLN* phospholamban, *TTN* titin, *TNNC1* troponin C, *TNNT2* troponin T2, *RBM20* RNA-binding motif protein 20, *SCN5A* sodium voltage-gated channel, α subunit 5

Most identified genes responsible for the DCM phenotype encode proteins within the myocytes. These are the sarcomere components, mitochondria, cytoskeleton, and desmosomal proteins. Genetic mutations implicated in DCM are predominantly composed of rare variants, making it challenging to diagnose a truly monogenic form of DCM [10]. A variant is considered pathogenic or likely pathogenic if there is a ≥ 90% likelihood of causing DCM [11]. The variable penetrance implies that although individuals may carry the disease-causing genotype, the DCM phenotype varies in clinical manifestation and severity. The DCM phenotype may only be evident in some candidates if the affected individual is further exposed to additional environmental or toxic insults such as alcohol. An international panel of clinical and scientific experts in DCM genetics has reviewed the available evidence on DCM genetics and identified twelve implicated genes from 8 gene ontologies as having a strong or definitive association with DCM [9]. Figure 1 depicts these “monogenic” genes classified according to their location in cardiac myocytes.

### Mechanism of cardiomyopathy based on causative genes

In up to 50% of cases, DCM is inherited in an autosomal dominant pattern [12]. Titin (*TTN*) has been reported as the primary causative gene [12]*.* Titin encodes the largest protein in the heart and functions as a spring that ties myosin to the Z-band in sarcomeres [13]. The second common gene implicated in the pathogenesis of DCM is the *LMNA* gene, which encodes protein lamins A and C. Mutations in this gene account for 5–10% of DCM and are responsible for the disruption of the chromatin organisation in dividing cells and signal transduction in non-dividing cells. Furthermore, *LMNA* DCM is often associated with atrioventricular blocks, atrial fibrillation, and ventricular arrhythmias. Patients with *LMNA* gene mutations may require primary prevention therapy, including a cardiac defibrillator device implantation, due to a 46% risk of sudden cardiac death [14, 15].

### Genetic studies in the African context

We conducted a systematic literature search in PubMed, Scopus, and Web of Science to identify original clinical research studies conducted in Africa that report genes and variants associated with dilated cardiomyopathy. The search string was used “Dilated cardiomyopathy AND genetics AND Africa”. We excluded studies reporting on genetic findings in peripartum cardiomyopathy. The Preferred Reporting Items For Systematic Reviews and Meta-Analyses (PRISMA) flow chart showing the selection of studies is available as a supplementary file.

The literature search yielded twelve studies (Table 2), with very few studies (*n* = 2) on familial or monogenic cases of DCM in Africa. Eight studies (67%) were conducted in South Africa, and the rest in Tunisia (*n* = 2), Morocco (*n* = 1), and Egypt (*n* = 1).

**Table 2:** Summary of twelve studies reporting on causal genetic mutations and variants in African patients with dilated cardiomyopathy

Reference	Year	Region	Sample size	Age (years)	Study population	Gene (type of study)	Results
Adadi et al. [16]	2018	Morocco	5 (family)	17–29	Four relatives and proband with DCM	*LMNA* heterozygous mutation c.1621C > T (p.Arg541Cys) (monogenic)	The phenotype of this mutation includes electrocardiographic abnormalities (nonspecific intraventricular block and pathological Q waves), regional LV akinesis/dyskinesis, and ventricular arrhythmias
Fish et al. [17]	2016	South Africa	95 (family)	-	8% European descent	Phospholamban (*PLN*) gene (monogenic)	The *PLN* c.25C > T mutation was part of this family’s disease-specific haplotype spanning a 5.15 Mb region Haplotype analysis revealed that this mutation occurred against a different haplotype background from the original North American family and was unlikely to have been inherited from a common ancestor
Sayed et al. [18]	2015	Egypt	80	4–14	Sixty HF children, 30 with HF due to idiopathic dilated cardiomyopathy (IDCM), 30 due to chronic renal failure (CRF) were compared to 20 healthy controls (HC)	The number of GT(n) repeats in the hemeoxygenase-1 (*HO-1*) gene promoter (association study)	The number of GT(n) repeats in the hemeoxygenase-1 gene promoter was increased in children with heart failure due to IDCM and chronic renal failure
Mahjoub et al. [19]	2010	Tunisia	187	19.9 ± 13.1 (DCM) 19.1 ± 9.8 (HC)	Primary DCM	Class II major histocompatibility complex (MHC) Genes: human leukocyte antigen (HLA)-DRB1 and -DQB1 alleles were analysed in 76 patients with primary DCM and 111 ethnically matched healthy controls (HC) (association study)	Increased frequencies of (i) HLA-DRB1*0401 [OR = 2*.*67, 95% CI (1.64–4.32) *P* < 0*.*001] (ii) HLA-DQB1*0302 [OR = 3*.*28, 95% CI (1.59–6.79) *P* = 0*.*001] and (iii) HLA-DQB1*0401 [OR = 6*.*26, 95% CI (1.73–22.57) *P* = 0*.*005] Alleles were found in patients with primary DCM compared with healthy controls. Individuals with HLA-DRB1*1301 [OR = 0*.*24, 95% CI (0.11–0.51) *P* < 0*.*001] and HLA-DQB1*0201 [OR = 0*.*49, 95% CI (0.32–0.77) *P* = 0*.*002] alleles have a protective effect against primary DCM. Two haplotypes were associated with an increased risk of primary DCM: (i) DRB1*0401/DQB1*0302 [OR = 4*.*53, 95% CI (1.74–11.78) *P* = 0*.*002] and (ii) DRB1*0401/DQB1*0401 [OR = 9*.*42, 95% CI (2.08–42.76) *P* = 0*.*004]
Mahjoub et al. [20]	2010	Tunisia	227	19.9 ± 13.1 (DCM) 19.1 ± 9.8 (HC)		Association of angiotensin-converting enzyme I/D polymorphism with the risk of dilated cardiomyopathy 76 patients with dilated cardiomyopathy were compared to 151 ethnically, age- and gender-matched controls (association study)	(i) The frequencies of the DD genotype and D allele were significantly higher in DCM patients as compared with controls and were associated with an increased risk of dilated cardiomyopathy ACE DD versus ID and II: OR = 3.05 (95%CI, 1.58–5.87; *p* = 0.001); D versus I: OR = 2.0 (95% CI: 1.35–2.97; *p* = 0.001) (ii) There was no association between the combined genotypes (DD + ID) or D allele and left ventricular end-diastolic diameter in dilated cardiomyopathy patients with severe and moderate clinical phenotypes (iii) DD genotype and D allele of angiotensin-converting enzyme I/D gene polymorphism are associated with an increased risk of dilated cardiomyopathy in a Tunisian population. However, it does not influence the cardiac phenotype severity
Shaboodien et al. [21]	2009	South Africa	30	35 (IQR: 27–41)	HIV-positive with DCM	The mitochondrial DNA (mtDNA) T16189C polymorphism, with a homopolymeric C-tract of 10–12 cytosines (association study)	The mtDNA T16189C variant with a homopolymeric C-tract was detected at a frequency of 26.7% (8/30) in the HIV-associated cardiomyopathy cases and 13.5% (5/37) in the HIV-positive controls. There was no significant difference between cases and controls (OR =2.33, 95% CI 0.67–8.06,* p* = 0.11)
Du Preez et al. [22]	2008	South Africa	71	48 ± 16 years	Black IDCM	A four-amino acid deletion was identified within the α2C-adrenergic receptor (α2CDel322-325) that, when homozygous increases the risk of heart failure in African-Americans nearly six-fold (association study)	No significant difference was observed between patients and controls in homozygosity for the α2CDel322-325 polymorphism or in allele and genotype frequencies. The frequency of the allele containing the deletion was 0.54 in cases and 0.53 in controls. The genotype frequencies in the patients were consistent with those of the controls (*p* = 0.56)
Woodiwiss et al. [23]	2008	South Africa	832	51.4 ± 0.4 (controls) 51.8 ± 0.8 (IDCM)	Black IDCM	β1-adrenoreceptor (AR) Gly389Arg and α2C-AR Del322-325 gene variants. The aim of the study was to assess the relationship between Gly389Arg and Del322-325 variants and the presence, severity, and progression of idiopathic dilated cardiomyopathy (IDCM) in 403 black South African patients and 429 controls (association study)	All patients and controls genotyped for the α2C-AR variant were homozygous for the Del322-325 (risk) allele. The Gly389Arg polymorphism was not associated with IDCM (control *n* = 429) (Arg389 allele homozygosity: OR = 1.03, 95% CI = 0.78 − 1.35), nor did it predict LVEF and cavity dimensions before or after therapy. In patients homozygous for the risk allele of the α2c-AR variant, the β1-AR variant neither increased the risk
Badenhorst et al. [24]	2007	South Africa	394	-		Arg16Gly and Gln27Glu polymorphisms of the b2- adrenoreceptor (b2-AR) gene. The study evaluated whether the Arg16Gly and Gln27Glu variants of the b2-AR gene predict left ventricular ejection fraction (LVEF) and LV end-diastolic diameter (LVEDD) in patients with idiopathic dilated cardiomyopathy (IDCM) before and six months after receiving standard medical therapy other than b-AR blockers	In heart failure, the functional Arg16Gly and Gln27Glu variants of the b2-AR gene has no independent effect on adverse structural remodelling and pump function
Khogali et al. [25]	2001	South Africa and White Europeans	679	Sporadic DCM: 93 white Europeans (17–69) and black South Africans (18–70) years	22 black DCM and 19 black controls. 93 white European DCM and 545 Controls	Mitochondrial DNA variant at position 16,189 (T16189C). The T16189C variant is a homoplasmic T to C transition at bp 16,189 in the non-coding control region close to the termination associated sequence and is present in 100% of an individual's mtDNA. It generates an uninterrupted homopolymeric C-tract, which causes the heteroplasmic length variation of mtDNA, presumably because of slippage during mtDNA replication (association study)	An association exists between T16189C with heteroplasmic length variation and sporadic dilated cardiomyopathy in two different populations, with an overall increased frequency in all cases combined (OR = 2·45, 95% CI 1·40–4·29, *p* = 0.002)
Candy et al. [26]	1999	South Africa	171	Mean age 52–53 years	IDCM, LVEF ≤ 40%	Insertion–deletion (ID) polymorphism of the angiotensin-converting enzyme (ACE) gene. DD genotype of the *ACE* gene (association study)	The DD genotype of the ACE gene is independently associated with both a reduced LV systolic performance and an increased LV cavity size in patients with IDCM
Tiago et al. [27]	2002	South Africa	382	Cases 53 ± 11 years. Controls 55 ± 9 years	Black IDCM	Variants of the ACE (insertion-deletion polymorphism), angiotensinogen (AGT; M235T polymorphism) and the aldosterone synthase (CYP11B2, C-344 T polymorphism) genes (response to therapy)	CYP11B2 gene variant predicts the variable improvement in LVEF that occurs after initiating heart failure therapy in IDCM

*ACE* angiotensin-converting enzyme, *AR* adrenoreceptor, *DCM* dilated cardiomyopathy, *DNA* deoxyribonucleic acid, *IDCM* idiopathic dilated cardiomyopathy, *HIV* human immunodeficiency virus, *HLA* human leucocyte antigen, *HO-1* heme oxygenase 1, *LMNA* lamin, *LV* left ventricular, *LVEF* left ventricular ejection fraction, *MHC* major histocompatibility complex

## A comprehensive approach to diagnosing dilated cardiomyopathy

### History taking and clinical examination

**Fig. 2:**
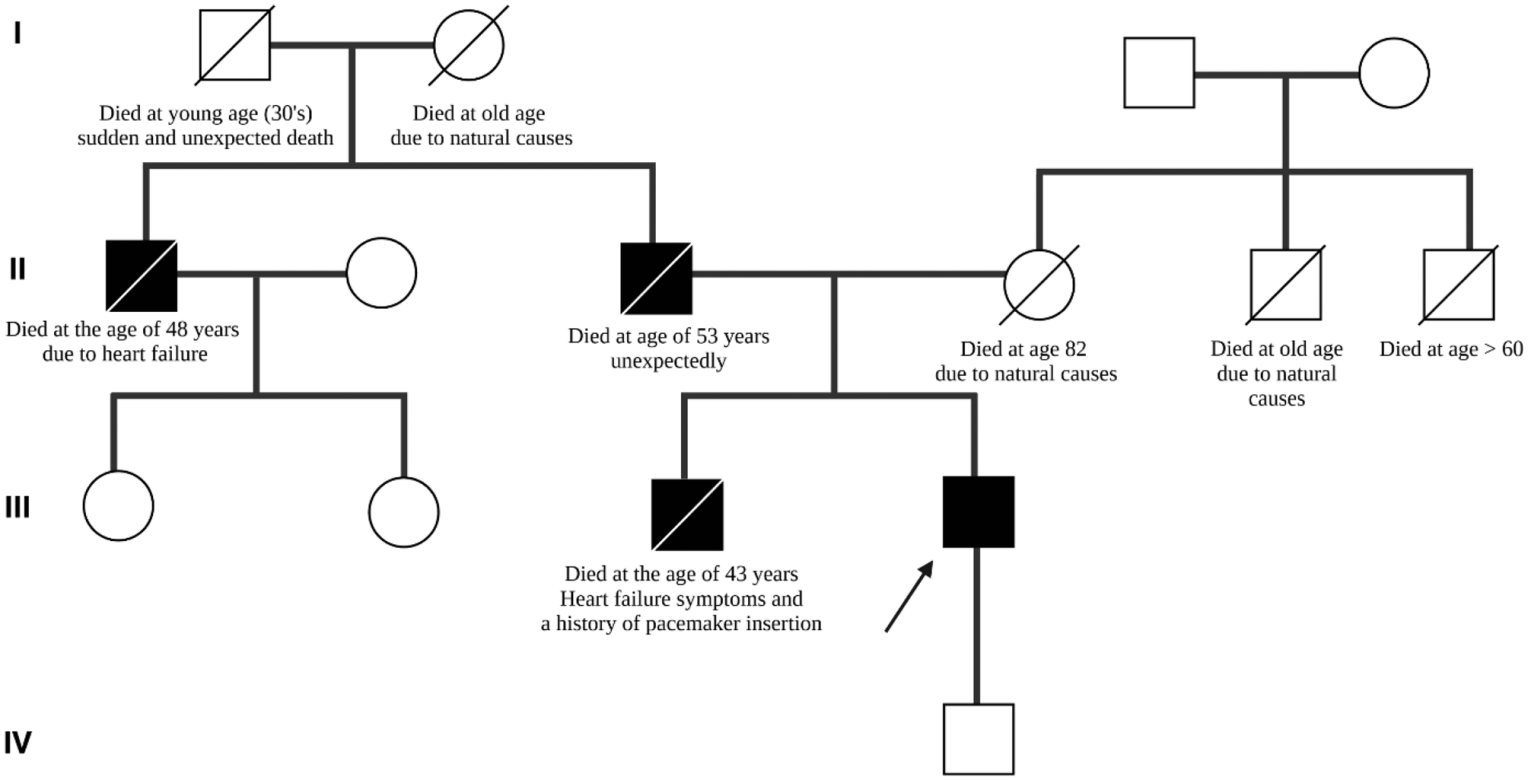
A four-generation family pedigree of a 62-year-old male (black arrow indicates proband) diagnosed with idiopathic dilated cardiomyopathy with a left ventricular ejection fraction (LVEF) of 30% at the age of 43. Squares shaded in black indicate male relatives with DCM. Females are represented with circles. Crossed-out circles and squares denote demised relatives

The first step in diagnosing DCM entails obtaining a comprehensive clinical history, physical examination, laboratory investigations, and imaging. History taking should include a three-generation family history and a pedigree [10]. Figure 2 demonstrates an example of a four-generation pedigree of a proband with IDCM. Clinical details related to the cohort, including the proband depicted in the pedigree, are described elsewhere [28].

Clinicians should enquire about a family history of sudden cardiac death, unexplained deaths before age 50, heart transplantation, and pacemaker insertion before age 55. Furthermore, a history of death due to unnatural causes or drownings should be elicited. If a family member died due to unnatural causes, post-mortem reports should be reviewed to exclude or confirm primary or secondary causes of death [10]. History taking should be followed by a thorough clinical examination that includes a neurological examination focused on identifying neuromuscular diseases such as muscular dystrophy, which may manifest as muscle wasting, contractures of the elbows, spine, and Achilles tendons [29].

### Electrocardiogram

Various electrocardiographic abnormalities may be found in patients with DCM, including a prolonged PR interval, evidence for ventricular hypertrophy, pathological Q waves, or bundle branch block [30–32]. In addition, poor prognostic factors on ECG include atrial fibrillation and a left bundle branch block [32].

### Echocardiogram

An echocardiogram is mandatory to confirm ventricular dilatation and calculate the LVEF, which may guide the selection of appropriate heart failure therapy. In addition, features of cardiac remodelling, which include increased left atrial size, functional mitral insufficiency, alteration of diastolic function, and involvement of other chambers, should be elicited as these features are also associated with an unfavourable prognosis [32, 33]. Speckle-tracking echocardiography may also help to identify asymptomatic patients with left ventricular systolic dysfunction before they manifest with an overt DCM phenotype [34].

### Laboratory tests

**Fig. 3:**
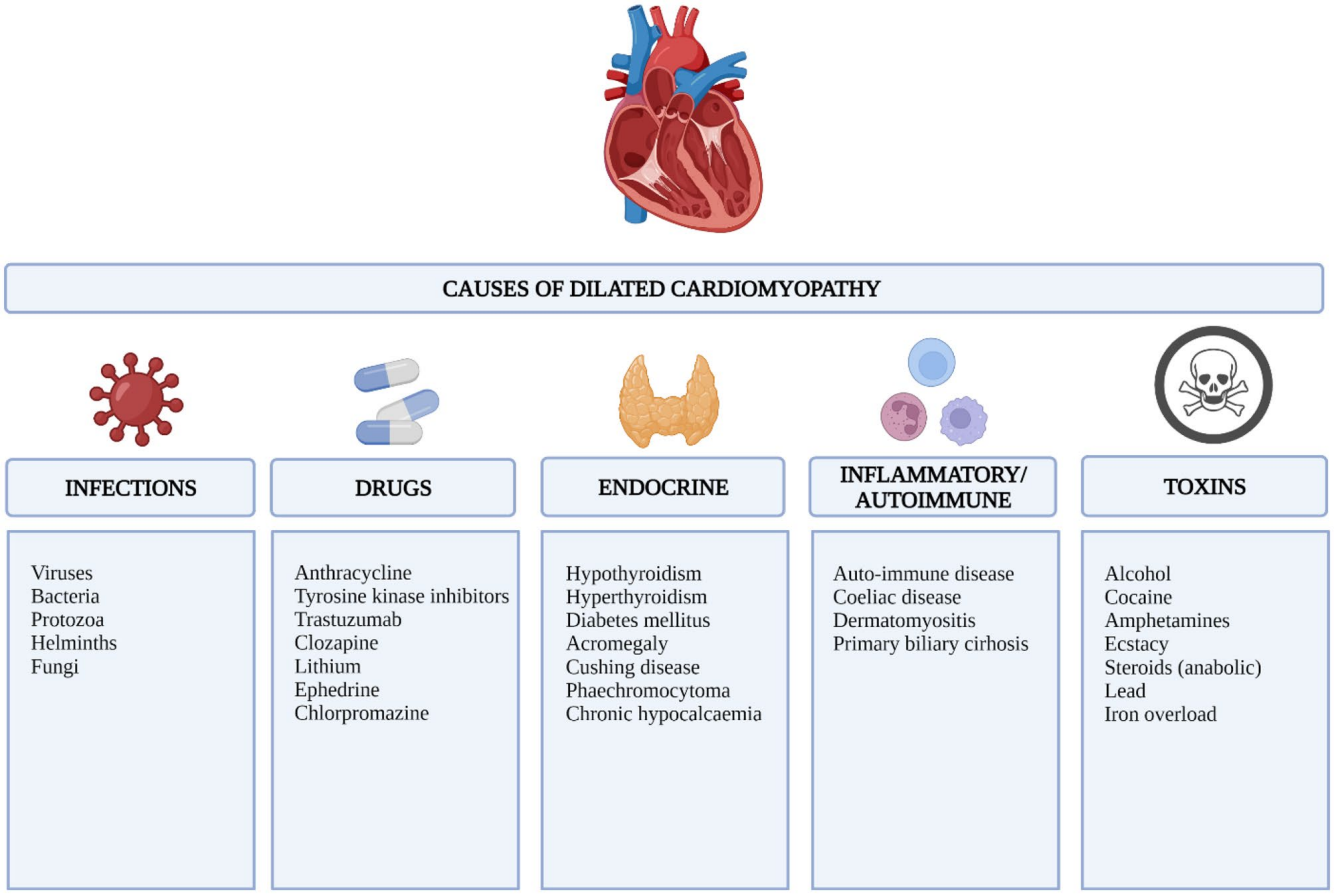
Causes of dilated cardiomyopathy that should be considered after excluding coronary artery disease

Laboratory biochemical tests are an integral part in the clinical workup of patients suspected to have IDCM. These may identify DCM’s endocrine, infectious, and haematological causes. Detecting micronutrient deficiencies such as selenium or thiamine may suggest malnutrition, which is strongly associated with alcohol misuse (Fig. 3). Abnormally high serial cardiac troponin and creatine kinase serum levels may indicate an acute myocyte injury due to myocarditis. Thyroid stimulating hormone and thyroxine levels should be assessed to exclude hypothyroidism or hyperthyroidism. Low calcium levels may indicate underlying chronic hypocalcaemia, while elevated iron levels may suggest underlying iron overload. When clinically suspected, bacterial and fungal infections should be excluded by performing blood cultures. Furthermore, a significantly elevated brain natriuretic peptide level has been reported to suggest a poor prognosis in patients with DCM [35–37].

### Coronary angiography

Coronary angiography is currently the gold standard for evaluating coronary epicardial vessels’ atherosclerotic disease. Therefore, all patients with DCM should ideally be referred for a diagnostic coronary angiogram to exclude coronary artery disease. Furthermore, this test is mandatory since the therapeutic approach varies in patients with ischaemic and non-ischaemic cardiomyopathy.

### Cardiovascular magnetic resonance imaging

Magnetic resonance imaging is crucial for excluding infiltrative conditions such as sarcoidosis and amyloidosis. In addition, the visualisation of gadolinium enhancement on late images may indicate underlying fibrosis. Several research studies have reported an association between the visualisation and burden of late gadolinium enhancement (LGE) and all-cause mortality in DCM [38–41].

### Endomyocardial biopsy

An endomyocardial biopsy provides a definite histological, immunohistochemistry, and molecular evaluation of myocardial tissue [11]. It is indicated in patients suspected to have acute myocarditis or chronic inflammatory cardiomyopathy. The evaluation of endomyocardial biopsy samples of DCM patients may show nonspecific histopathological signs such as hypertrophy and vacuolar changes of myocytes and fibrosis [42]. Although invasive, the risk of complications associated with an endocardial biopsy is low, with 11% of patients experiencing atrioventricular block [42].

Endomyocardial biopsies should be considered if the test will likely alter the therapeutic management of patients, mainly if conditions such as sarcoidosis, giant cell myocarditis, eosinophilic myocarditis, or hemochromatosis are considered differential diagnoses. Strategies that could improve access to myocardial biopsy, particularly in low- and middle-income countries (LMIC), include the implementation of referral pathway protocols that prioritise the performance of biopsies in these patients, ensuring that personnel are adequately trained, as well as increasing the number of available catheterisation laboratories.

### Genetic testing

Pre-test genetic counselling should be provided to DCM patients and their families. A detailed family history should be taken, and the possible findings and implications of genetic results should be explored during the counselling session. Defining terminology such as “pathogenic mutations, variants of uncertain significance and benign genetic variants” should be explained [8, 13]. Post-test counselling should focus on interpreting results, discussing reproductive risk, and the need for cascade family testing [13, 28].

Cardiomyopathy gene testing is still not widely available in most LMICs. However, diagnostic testing should be performed in carefully phenotyped patients with evidence of disease. In contrast, predictive testing is recommended in asymptomatic individuals (usually family members of an individual with a known DCM mutation) to predict the future risk of disease [43]. Currently available genetic tests involve sequencing a single gene or an individual variant, cardiomyopathy gene panel sequencing, whole-exome sequencing, and whole-genome sequencing [44].

Sequencing a single gene or individual variant should be considered a confirmatory test in a family member with a proband carrying a pathogenic/likely pathogenic variant detected through other techniques. Although this approach is cost-effective, it is not appropriate for diagnostic testing in a proband from another family with DCM where the causal mutation has not yet been identified [44, 45]. Cardiomyopathy gene panel sequencing involves sequencing the coding regions of several cardiomyopathy genes simultaneously in a single experiment. The diagnostic yield is higher. However, this test may not capture non-coding variants [13].

Whole-exome sequencing involves the analysis of a sequence of the entire coding region of the human genome. Limited non-coding regions may be included, but much of the non-coding DNA is not analysed. Whole-exome sequencing is not limited to genes previously linked with disease, thus enabling the potential to identify novel variants in new genes of interest. However, there is also a higher likelihood of identifying a variant of uncertain significance with whole-exome sequencing, and these usually need further investigation or careful explanation to patients as to their unknown implications [46]. In contrast, whole-genome sequencing captures both coding and non-coding variants, including deep intronic variants. Although more expensive, whole-genome sequencing will capture all variants and can be used to calculate polygenic risk scores for multifactorial causality of DCM and to report pharmacogenetic variants [47]. If a causal genetic mutation for monogenic DCM is identified, cascade screening of family members should be implemented, where a specific mutation rather than a gene panel is evaluated.

**Fig. 4:**
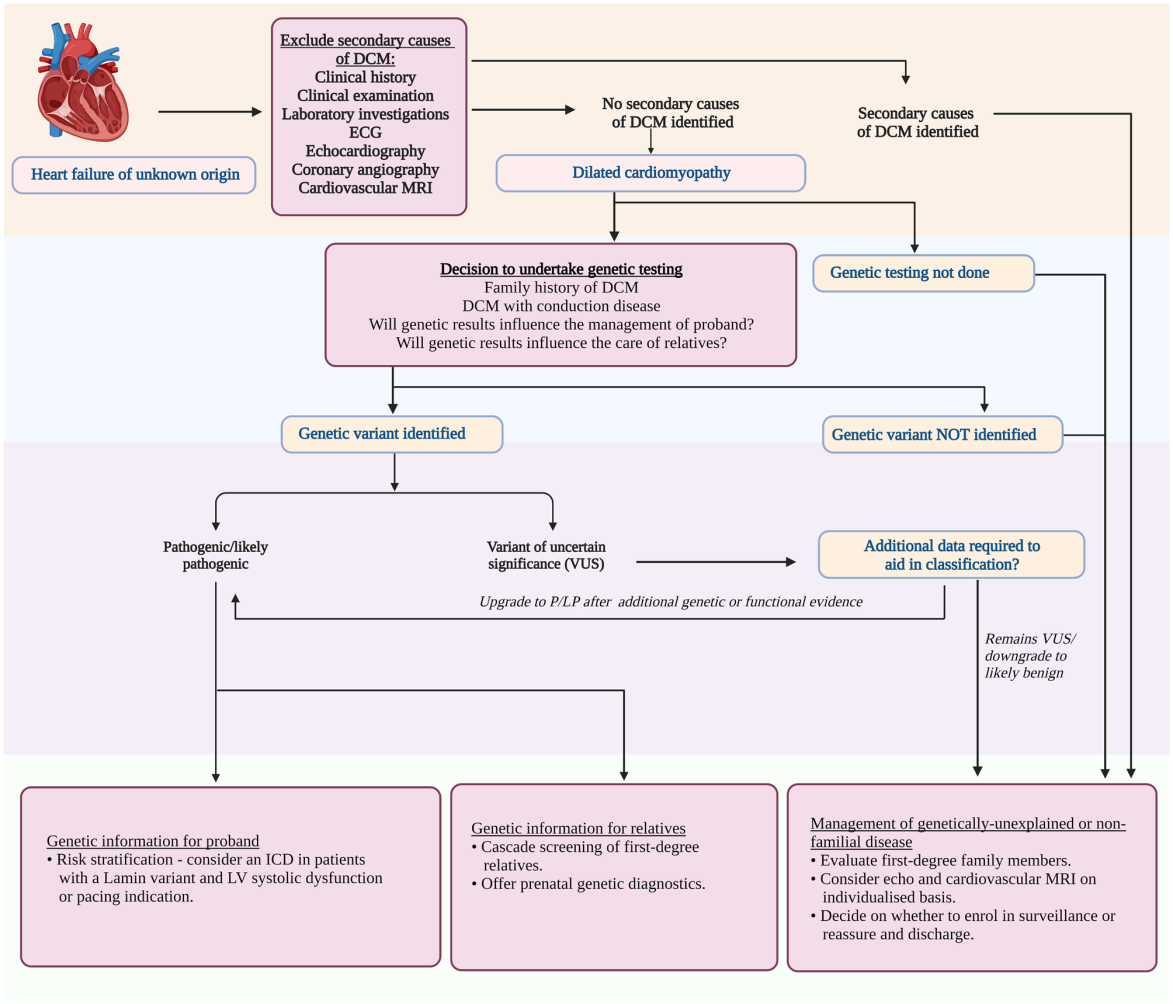
An approach to genetic testing in a proband with dilated cardiomyopathy. Diagram modified from Tayal et al. [10]. *DCM* dilated cardiomyopathy, *ECG* electrocardiogram, *ICD* implantable cardioverter defibrillator, *LP* likely pathogenic, *LV* left ventricular, *MRI* magnetic resonance imaging, *P* pathogenic, *VUS* variant of uncertain significance

Genetic testing within the African context is challenging, partly due to the diverse genetic makeup of populations within the continent, making the possibility of identifying a variant of uncertain significance higher [48]. Furthermore, currently available DCM gene panels could be limiting in the African context as the diagnostic yield may be lower since African-specific DCM-causing genes (should they be present) have not yet been identified. In addition, the current gene panels are based on DCM patients who are primarily not of African descent. Figure 4 summarises an approach to genetic testing in a proband with DCM.

### Differential diagnosis

Diagnosing DCM may be challenging in most LMICs and other remote regions without access to diagnostic modalities such as coronary angiography or cardiovascular MRI. In such scenarios, the diagnosis is generally made on clinical grounds. The higher prevalence of infectious diseases in sub-Saharan Africa makes viral myocarditis highly likely. As such, clinicians faced with patients presenting acutely with the clinical syndrome of heart failure should routinely evaluate and monitor serum troponins, creatine kinase-MB levels, and viral antibody titres to exclude viral myocarditis. The viral antibody titre tests may include coronavirus, enterovirus, HIV, cytomegalovirus, Epstein-Barr virus, adenovirus, human herpes virus 6, parvovirus B19, hepatitis, and influenza virus antibodies [49].

A genetic aetiology should be considered in females with idiopathic cardiomyopathy in the peripartum phase [50–53]. A discussion on the genetic basis of peripartum cardiomyopathy is beyond the scope of this manuscript, and the reader is referred to literature published elsewhere [54].

## Management of patients with genetic causes of dilated cardiomyopathy

The pharmacological management of a proband with a genetic cause for DCM is not different from that of any individual with non-genetic causes of DCM. It is guided by the presence of heart failure symptoms, the baseline LVEF, and the presence or risk for potentially lethal arrhythmias. Pharmacological therapy for DCM is mainly based on neurohumoral blockade [55]. Diuretics and inotropes may be considered in the acute phase in patients with congestive heart failure complicated by cardiogenic shock. Agents such as beta-blockers, angiotensin-converting enzyme inhibitors (ACE-I) or angiotensin receptor blockers in ACE-I intolerant individuals, angiotensin receptor neprilysin inhibitors (ARNI), mineralocorticoid antagonists (MRA), and more recently the sodium-glucose cotransporter-2 (SGLT2) inhibitors are currently considered essential foundational therapies in the management of heart failure with reduced ejection fraction [37].

Mutations in the *LMNA* gene have been linked to atrioventricular block and atrial or ventricular arrhythmias [56, 57]. To prevent lethal ventricular arrhythmias, implantable cardioverter defibrillators should be considered, particularly in patients who have survived a malignant ventricular tachyarrhythmia or those presenting with symptomatic ventricular tachycardia [15]. Unfortunately, device therapy is still not widely available in most LMICs due to its high cost.

### Targeted therapies

Several strategies that address genetic abnormalities in patients with DCM are available. These methods include targeting specific single mutations, exon skipping, and gene replacement targeting all mutations at once by gene transfer of the full-length complementary DNA [55]. Also, newer molecules such as EMD 57,033 bind in the same region of myosin, increasing the rate of ATP binding, hydrolysis, and actin interactions. Furthermore, CK-1827452, a molecule that accelerates the transition of the actin-myosin complex from weakly to strong bound, is being investigated for potential clinical use [58]. Also, there is an ongoing randomised, double-blind placebo-controlled trial evaluating the efficacy of ARRY-371797, an inhibitor of the p38α MAPK pathway in symptomatic patients with DCM due to a mutation of the gene encoding the lamin A/C protein [59].

## Future directions and recommendations

There is an urgent need to establish registries for IDCM/DCM patients and their families in Africa to facilitate the investigation of a genetic basis for IDCM in patients of African ancestry [60]. This should be coupled with the creation of African genome banks to enable the ease of determining the pathogenicity of VUS against a suitable reference population. Establishing African genomic consortia such as the Human Heredity and Health in Africa (H3Africa) Consortium promises to make these goals a reality [61].

## Conclusion

IDCM remains a common disease in many African regions and continues to cause significant morbidity and mortality in young patients. Given that Africa has remarkable genetic diversity, a study of the genetic aetiology of this condition in an African setting is likely to yield novel insights and could be of clinical relevance in diagnosis and treatment. This highlights the need for improved access to IDCM genetic testing. Once widely implemented, the term “idiopathic dilated cardiomyopathy” should only be reserved for patients with a comprehensive clinical workup that includes genetic testing that fails to identify a plausible cause for DCM.

## Supplementary Information

Below is the link to the electronic supplementary material.

Supplementary file1 (DOCX 39 KB)

## Data Availability

Not applicable.

## References

[1] Agbor VN, Essouma M, Ntusi NaB, Nyaga UF, Bigna JJ, Noubiap JJ (2018) Heart failure in sub-Saharan Africa: a contemporaneous systematic review and meta-analysis. Int J Cardiol 257. 10.1016/j.ijcard.2017.12.048 10.1016/j.ijcard.2017.12.048 29506693

[2] Steenekamp JH, Simson IW, Theron W (1992) Cardiovascular causes of death at Tshepong Hospital in 1 year, 1989–1990. A necropsy study. S Afr Med J 81(3) 1734552

[3] Stewart S, Carrington M, Pretorius S, Methusi P, Sliwa K (2011) Standing at the crossroads between new and historically prevalent heart disease: effects of migration and socio-economic factors in the Heart of Soweto cohort study. Eur Heart J 32(4). 10.1093/eurheartj/ehq439 10.1093/eurheartj/ehq439 21163850

[4] Dokainish H, Teo K, Zhu J, Roy A, Alhabib KF, Elsayed A et al (2013) Heart failure in Africa, Asia, the Middle East and South America: the INTER-CHF study. Int J Cardiol 204. 10.1016/j.ijcard.2015.11.183 10.1016/j.ijcard.2015.11.183 26657608

[5] Callender T, Woodward M, Roth G, Farzadfar F, Lemarie JC, Gicquel S et al (2014) Heart failure care in low- and middle-income countries: a systematic review and meta-analysis. PLoS Med 11(8). 10.1371/journal.pmed.1001699 10.1371/journal.pmed.1001699 PMC4130667 25117081

[6] Elliott P, Andersson B, Arbustini E, Bilinska Z, Cecchi F, Charron P et al (2008) Classification of the cardiomyopathies: a position statement from the European Society Of Cardiology Working Group on Myocardial and Pericardial Diseases. Eur Heart J 29(2). 10.1093/eurheartj/ehm342 10.1093/eurheartj/ehm342 17916581

[7] Fatkin D, Johnson R, Mcgaughran J, Weintraub RG, Atherton JJ, Group CGCW (2017) Position statement on the diagnosis and management of familial dilated cardiomyopathy. Heart Lung Circ 26(11). 10.1016/j.hlc.2017.04.021 10.1016/j.hlc.2017.04.021 28655534

[8] Rosenbaum AN, Agre KE, Pereira NL (2020) Genetics of dilated cardiomyopathy: practical implications for heart failure management. Nat Rev Cardiol 17(5). 10.1038/s41569-019-0284-0 10.1038/s41569-019-0284-0 31605094

[9] Jordan E, Peterson L, Ai T, Asatryan B, Bronicki L, Brown E et al (2021) Evidence-based assessment of genes in dilated cardiomyopathy. Circulation 144(1). 10.1161/CIRCULATIONAHA.120.053033 10.1161/CIRCULATIONAHA.120.053033 PMC8247549 33947203

[10] Tayal U, Ware JS, Lakdawala NK, Heymans S, Prasad SK (2021) Understanding the genetics of adult-onset dilated cardiomyopathy: what a clinician needs to know. Eur Heart J 42(24). 10.1093/eurheartj/ehab286 10.1093/eurheartj/ehab286 PMC8216730 34153989

[11] Schultheiss HP, Fairweather D, Caforio ALP, Escher F, Hershberger RE, Lipshultz SE et al (2019) Dilated cardiomyopathy. Nat Rev Dis Primers 5(1). 10.1038/s41572-019-0084-1 10.1038/s41572-019-0084-1 PMC7096917 31073128

[12] Shaboodien G, Spracklen TF, Kamuli S, Ndibangwi P, Van Niekerk C, Ntusi NaB (2020) Genetics of inherited cardiomyopathies in Africa. Cardiovascular diagnosis and therapy 10(2). 10.21037/cdt.2019.10.03 10.21037/cdt.2019.10.03 PMC7225421 32420109

[13] Mcnally EM, Mestroni L. Dilated cardiomyopathy: genetic determinants and mechanisms. Circ Res. 2017;121(7). 10.1161/CIRCRESAHA.116.309396 10.1161/CIRCRESAHA.116.309396 PMC5626020 28912180

[14] Van Berlo JH, De Voogt WG, Van Der Kooi AJ, Van Tintelen JP, Bonne G, Yaou RB et al (2005) Meta-analysis of clinical characteristics of 299 carriers of LMNA gene mutations: do lamin A/C mutations portend a high risk of sudden death? J Mol Med (Berl) 83(1). 10.1007/s00109-004-0589-1 10.1007/s00109-004-0589-1 15551023

[15] Ciarambino T, Menna G, Sansone G, Giordano M (2021) Cardiomyopathies: an overview. Int J Mol Sci 22(14). 10.3390/ijms22147722 10.3390/ijms22147722 PMC8303989 34299342

[16] Adadi N, Radi FZ, Lahrouchi N, Hara L, Ratbi I, Elalaoui SC et al (2018) Inherited dilated cardiomyopathy in a large Moroccan family caused by LMNA mutation. Anatol J Cardiol 20(1). 10.14744/AnatolJCardiol.2018.69639 10.14744/AnatolJCardiol.2018.69639 PMC6237802 29952368

[17] Fish M, Shaboodien G, Kraus S, Sliwa K, Seidman CE, Burke MA et al (2016) Mutation analysis of the phospholamban gene in 315 South Africans with dilated, hypertrophic, peripartum and arrhythmogenic right ventricular cardiomyopathies. Sci Rep 6. 10.1038/srep22235 10.1038/srep22235 PMC4808831 26917049

[18] Sayed S, Idriss NK, Blann A, Sayyed HG, Raafat DM, Fouad D et al (2015) The number of GT(n) repeats in the hemeoxygenase-1 gene promoter is increased in pediatric heart failure but is unrelated to renal, antioxidant and anti-inflammatory markers. Pediatric cardiology 36(6). 10.1007/s00246-015-1146-0 10.1007/s00246-015-1146-0 25822459

[19] Mahjoub S, Mehri S, Ghazouani E, Ouarda F, Boussada R, Zaroui A et al (2010) HLA class II polymorphisms in Tunisian patients with dilated cardiomyopathy. Tissue Antigens 75(6). 10.1111/j.1399-0039.2009.01432.x 10.1111/j.1399-0039.2009.01432.x 20136773

[20] Mahjoub S, Mehri S, Ourda F, Boussaada R, Zouari B, Ben Arab S (2011) [Epidemiological study of the idiopathic dilated cardiomyopathy in Tunisia]. Ann Cardiol Angeiol (Paris) 60(4). 10.1016/j.ancard.2011.04.006 10.1016/j.ancard.2011.04.006 21663894

[21] Shaboodien G, Engel ME, Syed FF, Poulton J, Badri M, Mayosi BM (2009) The mitochondrial DNA T16189C polymorphism and HIV-associated cardiomyopathy: a genotype-phenotype association study. BMC Med Genet 10. 10.1186/1471-2350-10-37 10.1186/1471-2350-10-37 PMC2679724 19397801

[22] Du Preez J, Matolweni LO, Greenberg J, Mntla P, Adeyemo AA, Mayosi BM (2018) The α2CDel322–325 adrenergic receptor polymorphism is not associated with heart failure due to idiopathic dilated cardiomyopathy in black Africans PMC3975310 18320080

[23] Woodiwiss AJ, Badenhorst D, Sliwa K, Brooksbank R, Essop R, Sareli P et al (2008) β1- and α2c-adrenoreceptor variants as predictors of clinical aspects of dilated cardiomyopathy in people of African ancestry PMC3971767 18776959

[24] Badenhorst D, Norton GR, Sliwa K, Brooksbank R, Essop R, Sareli P et al (2007) Impact of beta2-adrenoreceptor gene variants on cardiac cavity size and systolic function in idiopathic dilated cardiomyopathy. Pharmacogenomics J 7(5). 10.1038/sj.tpj.6500426 10.1038/sj.tpj.6500426 17117186

[25] Khogali SS, Mayosi BM, Beattie JM, Mckenna WJ, Watkins H, Poulton J (2001) A common mitochondrial DNA variant associated with susceptibility to dilated cardiomyopathy in two different populations. Lancet (London, England) 357(9264). 10.1016/S0140-6736(00)04422-6 10.1016/S0140-6736(00)04422-6 11418155

[26] Candy GP, Skudicky D, Mueller UK, Woodiwiss AJ, Sliwa K, Luker F et al (1999) Association of left ventricular systolic performance and cavity size with angiotensin-converting enzyme genotype in idiopathic dilated cardiomyopathy. The American journal of cardiology 83(5). 10.1016/s0002-9149(98)00981-3 10.1016/s0002-9149(98)00981-3 10080429

[27] Tiago AD, Badenhorst D, Skudicky D, Woodiwiss AJ, Candy GP, Brooksbank R et al (2002) An aldosterone synthase gene variant is associated with improvement in left ventricular ejection fraction in dilated cardiomyopathy. Cardiovasc Res 54(3). 10.1016/s0008-6363(02)00281-x 10.1016/s0008-6363(02)00281-x 12031704

[28] Bailly C, Henriques S, Tsabedze N, Krause A (2019) Role of family history and clinical screening in the identification of families with idiopathic dilated cardiomyopathy in Johannesburg, South Africa. S Afr Med J 109(9). 10.7196/SAMJ.2019.v109i9.13936 10.7196/SAMJ.2019.v109i9.13936 31635593

[29] Brown CA, Lanning RW, Mckinney KQ, Salvino AR, Cherniske E, Crowe CA et al (2001) Novel and recurrent mutations in lamin A/C in patients with Emery-Dreifuss muscular dystrophy. Am J Med Genet 102(4). 10.1002/ajmg.1463 10.1002/ajmg.1463 11503164

[30] Dec GW, Fuster V (1994) Idiopathic dilated cardiomyopathy. New England Journal of Medicine 331(23). 10.1056/NEJM199412083312307 10.1056/NEJM199412083312307 7969328

[31] Lakdawala NK, Winterfield JR, Funke BH (2013) Dilated cardiomyopathy. Circ Arrhythm Electrophysiol 6(1). 10.1161/CIRCEP.111.962050 10.1161/CIRCEP.111.962050 PMC3603701 23022708

[32] Merlo M, Cannata A, Gobbo M, Stolfo D, Elliott PM, Sinagra G (2018) Evolving concepts in dilated cardiomyopathy. Eur J Heart Fail 20(2). 10.1002/ejhf.1103 10.1002/ejhf.1103 29271570

[33] Japp AG, Gulati A, Cook SA, Cowie MR, Prasad SK (2016) The diagnosis and evaluation of dilated cardiomyopathy. J Am Coll Cardiol 67(25). 10.1016/j.jacc.2016.03.590 10.1016/j.jacc.2016.03.590 27339497

[34] Murtaza G, Virk HUH, Khalid M, Rahman Z, Sitwala P, Schoondyke J et al (2017) Role of speckle tracking echocardiography in dilated cardiomyopathy: a review. Cureus 9(6). 10.7759/cureus.1372 10.7759/cureus.1372 PMC5519311 28744419

[35] Gardner RS, Ozalp F, Murday AJ, Robb SD, Mcdonagh TA (2003) N-terminal pro-brain natriuretic peptide. A new gold standard in predicting mortality in patients with advanced heart failure. Eur Heart J 24(19). 10.1016/j.ehj.2003.07.005 10.1016/j.ehj.2003.07.005 14522568

[36] Kim H, Cho YK, Jun DH, Nam CW, Han SW, Hur SH et al (2008) Prognostic implications of the NT-ProBNP level and left atrial size in non-ischemic dilated cardiomyopathy. Circ J 72(10). 10.1253/circj.cj-07-1087 10.1253/circj.cj-07-1087 18728335

[37] Mcdonagh TA, Metra M, Adamo M, Gardner RS, Baumbach A, Bohm M et al (2021) 2021 ESC Guidelines for the diagnosis and treatment of acute and chronic heart failure. Eur Heart J 42(36). 10.1093/eurheartj/ehab368 10.1093/eurheartj/ehab368 34447992

[38] Becker MaJ, Cornel JH, Van De Ven PM, Van Rossum AC, Allaart CP, Germans T (2018) The prognostic value of late gadolinium-enhanced cardiac magnetic resonance imaging in nonischemic dilated cardiomyopathy: a review and meta-analysis. JACC Cardiovasc Imaging 11(9). 10.1016/j.jcmg.2018.03.006 10.1016/j.jcmg.2018.03.006 29680351

[39] Di Marco A, Anguera I, Schmitt M, Klem I, Neilan TG, White JA et al (2017) Late Gadolinium enhancement and the risk for ventricular arrhythmias or sudden death in dilated cardiomyopathy: systematic review and meta-analysis. JACC Heart Fail 5(1). 10.1016/j.jchf.2016.09.017 10.1016/j.jchf.2016.09.017 28017348

[40] Pi SH, Kim SM, Choi JO, Kim EK, Chang SA, Choe YH et al (2018) Prognostic value of myocardial strain and late gadolinium enhancement on cardiovascular magnetic resonance imaging in patients with idiopathic dilated cardiomyopathy with moderate to severely reduced ejection fraction. J Cardiovasc Magn Reson 20(1). 10.1186/s12968-018-0466-7 10.1186/s12968-018-0466-7 PMC6001169 29898740

[41] Zhang K, Wang W, Zhao S, Katz SD, Iervasi G, Gerdes AM et al (2018) Long-term prognostic value of combined free triiodothyronine and late gadolinium enhancement in nonischemic dilated cardiomyopathy. Clin Cardiol 41(1). 10.1002/clc.22858 10.1002/clc.22858 PMC6489825 29360143

[42] Seferovic PM, Tsutsui H, Mcnamara DM, Ristic AD, Basso C, Bozkurt B et al (2021) Heart Failure Association, Heart Failure Society of America, and Japanese Heart Failure Society position statement on endomyocardial biopsy. J Card Fail 27(7). 10.1016/j.cardfail.2021.04.010

[43] Evans JP, Skrzynia C, Burke W (2001) The complexities of predictive genetic testing. BMJ 322(7293). 10.1136/bmj.322.7293.1052 10.1136/bmj.322.7293.1052 PMC1120190 11325775

[44] Hershberger RE, Givertz MM, Ho CY, Judge DP, Kantor PF, Mcbride KL et al (2018) Genetic evaluation of cardiomyopathy: a clinical practice resource of the American College of Medical Genetics and Genomics (ACMG). Genet Med 20(9). 10.1038/s41436-018-0039-z 10.1038/s41436-018-0039-z 29904160

[45] Bozkurt B, Colvin M, Cook J, Cooper LT, Deswal A, Fonarow GC et al (2016) Current diagnostic and treatment strategies for specific dilated cardiomyopathies: a scientific statement from the American Heart Association. Circulation. 134(23). 10.1161/CIR.0000000000000455 10.1161/CIR.0000000000000455 27832612

[46] Ramchand J, Wallis M, Macciocca I, Lynch E, Farouque O, Martyn M et al (2020) Prospective evaluation of the utility of whole exome sequencing in dilated cardiomyopathy. J Am Heart Assoc 9(2). 10.1161/JAHA.119.013346 10.1161/JAHA.119.013346 PMC7033851 31931689

[47] Minoche AE, Horvat C, Johnson R, Gayevskiy V, Morton SU, Drew AP et al (2019) Genome sequencing as a first-line genetic test in familial dilated cardiomyopathy. Genet Med 21(3). 10.1038/s41436-018-0084-7 10.1038/s41436-018-0084-7 PMC7271716 29961767

[48] Krause A (2019) New genetic testing technologies: advantages and limitations. S Afr Med J 109(4). 10.7196/SAMJ.2019.v109i4.13990

[49] Tschope C, Ammirati E, Bozkurt B, Caforio ALP, Cooper LT, Felix SB et al (2021) Myocarditis and inflammatory cardiomyopathy: current evidence and future directions. Nat Rev Cardiol 18(3). 10.1038/s41569-020-00435-x 10.1038/s41569-020-00435-x PMC7548534 33046850

[50] Horne BD, Rasmusson KD, Alharethi R, Budge D, Brunisholz KD, Metz T et al (2011) Genome-wide significance and replication of the chromosome 12p11.22 locus near the PTHLH gene for peripartum cardiomyopathy. Circ Cardiovasc Genet 4(4). 10.1161/CIRCGENETICS.110.959205 10.1161/CIRCGENETICS.110.959205 21665988

[51] Morales A, Painter T, Li R, Siegfried JD, Li D, Norton N et al (2010) Rare variant mutations in pregnancy-associated or peripartum cardiomyopathy. Circulation 121(20). 10.1161/CIRCULATIONAHA.109.931220 10.1161/CIRCULATIONAHA.109.931220 PMC2900861 20458009

[52] Sheppard R, Hsich E, Damp J, Elkayam U, Kealey A, Ramani G et al (2016) GNB3 C825T Polymorphism and myocardial recovery in peripartum cardiomyopathy: results of the multicenter investigations of pregnancy-associated cardiomyopathy study. Circ Heart Fail 9(3). 10.1161/CIRCHEARTFAILURE.115.002683 10.1161/CIRCHEARTFAILURE.115.002683 PMC4770544 26915373

[53] Ware JS, Li J, Mazaika E, Yasso CM, Desouza T, Cappola TP et al (2016) Shared genetic predisposition in peripartum and dilated cardiomyopathies. N Engl J Med 374(3). 10.1056/NEJMoa1505517 10.1056/NEJMoa1505517 PMC4797319 26735901

[54] Goli R, Li J, Brandimarto J, Levine LD, Riis V, Mcafee Q et al (2021) Genetic and phenotypic landscape of peripartum cardiomyopathy. Circulation 143(19). 10.1161/CIRCULATIONAHA.120.052395 10.1161/CIRCULATIONAHA.120.052395 PMC8113098 33874732

[55] Verdonschot JaJ, Hazebroek MR, Ware JS, Prasad SK, Heymans SRB (2019) Role of targeted therapy in dilated cardiomyopathy: the challenging road toward a personalized approach. J Am Heart Assoc 8(11). 10.1161/JAHA.119.012514 10.1161/JAHA.119.012514 PMC6585365 31433726

[56] Crasto S, My I, Di Pasquale E (2020) The Broad Spectrum of LMNA Cardiac diseases: from molecular mechanisms to clinical phenotype. Front Physiol 11. 10.3389/fphys.2020.00761 10.3389/fphys.2020.00761 PMC7349320 32719615

[57] Ollila L, Nikus K, Holmstrom M, Jalanko M, Jurkko R, Kaartinen M et al (2017) Clinical disease presentation and ECG characteristics of LMNA mutation carriers. Open Heart 4(1). 10.1136/openhrt-2016-000474 10.1136/openhrt-2016-000474 PMC5255551 28123761

[58] Repetti GG, Toepfer CN, Seidman JG, Seidman CE (2019) Novel therapies for prevention and early treatment of cardiomyopathies. Circ Res 124(11). 10.1161/CIRCRESAHA.119.313569 10.1161/CIRCRESAHA.119.313569 PMC7092753 31120825

[59] Pfizer (2021) A study of ARRY-371797 (PF-07265803) in patients with symptomatic dilated cardiomyopathy due to a lamin A/C gene mutation. Available from: https://www.pfizerclinicaltrials.com/find-a-trial/nct03439514.

[60] Kraus SM, Shaboodien G, Francis V, Laing N, Cirota J, Chin A et al (2021) Rationale and design of the African Cardiomyopathy and Myocarditis Registry Program: the IMHOTEP study. Int J Cardiol 333. 10.1016/j.ijcard.2021.02.026 10.1016/j.ijcard.2021.02.026 33607192

[61] Peprah E, Wiley K, Sampson U, Narula J (2017) A New Age for African-Driven Genomics Research: Human Heredity and Health in Africa (H3Africa). Glob Heart 12(2). 10.1016/j.gheart.2017.05.003 10.1016/j.gheart.2017.05.003 28867289

