# Supplementary material for: The genetic basis for adult-onset idiopathic dilated cardiomyopathy in people of African descent
Source: Heart Fail Rev. 2023 Mar 14;28(4):879–92. doi: 10.1007/s10741-023-10302-9 (PMC10011790; doi:10.1007/s10741-023-10302-9)
Supplement: Supplementary file 1 — Supplementary file1 (DOCX 39 KB) [file 10741_2023_10302_MOESM1_ESM.docx]

**Supplementary file:** PRISMA flow chart for the selection of studies included in Table 2

**Identification of studies via databases**

Records removed *before screening*:

Duplicate records removed (n = 4)

Records marked as ineligible (n =69)

Records identified from:

PubMed (n =49)

Scopus (n=21)

Web of Science (n=26)

**Identification**

Records screened

(n = 23)

Records excluded

Review articles (n = 8)

Reports sought for retrieval

(n =15)

Reports not retrieved

(n =0)

**Screening**

Reports excluded:

Peripartum cardiomyopathy (n = 1)

No African patients (n =1)

Unrelated to DCM (n =1)

Reports assessed for eligibility

(n =15)

Studies included in review

(n =12)

**Included**
